# Mycobacteriophages as Genomic Engineers and Anti-infective Weapons

**DOI:** 10.1128/mBio.00632-21

**Published:** 2021-05-18

**Authors:** Mark R. Sullivan, Eric J. Rubin, Charles L. Dulberger

**Affiliations:** a Department of Immunology and Infectious Diseases, Harvard T. H. Chan School of Public Health, Boston, Massachusetts, USA; b Department of Microbiology, Harvard Medical School, Boston, Massachusetts, USA; c Department of Molecular and Cellular Biology, Harvard University, Boston, Massachusetts, USA

**Keywords:** *Mycobacterium abscessus*, bacterial genetics, bacteriophage genetics, bacteriophage therapy, molecular genetics, phage susceptibility

## Abstract

Mycobacterium abscessus (Mab) is an emerging pathogen that is highly tolerant to current antibiotic therapies, and the current standard of care has a high failure rate. Mycobacteriophages represent a promising alternative treatment that have the potential to kill Mab with few side effects. However, the repertoire of phages that infect Mab is limited, and little is understood about the determinants of phage susceptibility in mycobacteria. Two studies from the Hatfull group (R. M. Dedrick, B. E. Smith, R. A. Garlena, D. A. Russell, et al., mBio 12:e03431-20, 2021, https://doi.org/10.1128/mBio.03431-20, and R. M. Dedrick, H. G. Aull, D. Jacobs-Sera, R. A. Garlena, et al., mBio 12:e03441-20, 2021, https://doi.org/10.1128/mBio.03441-20) shed new light on the natural phage complement of Mab and provide some of the first insights into what factors might drive susceptibility to these phages. These studies not only lay the groundwork for therapeutic development of more effective phage therapy in Mab but also provide a foothold for studying how mobile elements such as phages and plasmids impact Mab biology and evolution.

## COMMENTARY

Phages have multiple roles in the lives of bacteria. While their raison d’être is to ultimately make more phages, their ability to carry DNA and mediate chromosomal rearrangements makes them important genomic engineers. And, of course, they can kill their hosts, allowing them to potentially be harnessed to fight bacterial infections. Two papers from the Hatfull lab cover both aspects of phage biology for the pathogen Mycobacterium abscessus (Mab) and help show the interrelationship of phages as tools for both infection and evolution.

Mab infections are particularly difficult to treat. These bacteria are inherently tolerant to many antibiotics and can acquire resistance during the course of chronic infection. Recently, there has been some success in developing an alternative treatment, using mycobacteriophages, a strategy that had apparent success in a patient with cystic fibrosis (1). Unfortunately, the repertoire of known phages that can infect and kill Mab is currently limited. The two studies published in *mBio* from Dedrick et al. (2, 3) serve as a step toward expanding the arsenal of phages available for therapeutic applications. This work represents a tour de force in clinical isolate handling and characterization—Mab isolates from 78 patients living in 11 different countries were put through a gambit of testing in order to better understand their phage susceptibility profiles, pathways of acquired resistance, and their sprawling genetics. In some cases, this knowledge was used to generate personalized phage therapies for people in dire need with drug-resistant infections. This is a tremendous resource that has been enabled by a network of students in the SEA-PHAGES program, who have collected, characterized, and smartly named mycobacteriophages from around the world (4).

Unfortunately, the authors were not able to find appropriate phages for all Mab strains. This is not surprising, as Mab clinical isolates are incredibly diverse; only about one-quarter of identified Mab genes are a part of the core genome that is present in all isolates of the species (5). This high level of diversity is generated in large part by horizontal gene transfer (6–8). Bacteriologists have long appreciated that phenotypic properties could be exchanged between bacteria. We now understand that a substantial proportion of this exchange is mediated by bacteriophages, which are able to package segments of the host genome and directly transfer that DNA to a new bacterium, as well as serve as a nexus for genomic rearrangements (9). Much work has focused on the transfer of clinically important genes that confer virulence or antibiotic resistance to the recipient bacteria (10, 11). These events can allow bacteria to more rapidly adapt to their environment than would be possible through chromosomal mutation and can lead to the advent of highly pathogenic bacteria (12). In Mab, virulence and antibiotic susceptibility can vary dramatically between clinical isolates (13–17), and the factors that shape these characteristics are only partially understood. The broad diversity of virulence-associated prophage genes as well as plasmid-derived transporters discovered by Dedrick et al. provide a critical entry point into unraveling the factors that determine Mab infectiousness and drug tolerance.

The logical focus on horizontal transfer of virulence and antibiotic resistance genes has perhaps obscured the broader impact of phage-mediated gene transfer on bacterial biology. Between 0.5 and 10% of the Mycobacterium tuberculosis genome may have been acquired by evolutionarily recent horizontal gene transfer (18), and similar trends are observed across nontuberculous mycobacteria (NTM). Further, in Mab, phage-mediated gene transfer appears to occur on a faster timescale than chromosomal mutation, suggesting that phages likely play a major role in shaping the Mab genome beyond simply transferring virulence and antibiotic tolerance genes (5, 6). For instance, in mycobacteria, the most highly represented category of horizontally acquired genes is in metabolism (7, 10), which may reflect the importance of rapid acquisition of genes in order to adapt to novel nutrient environments. The sheer abundance of prophages identified by Dedrick et al. across Mab genomes further underscores the central role phages play in determining the content and large-scale structure of the genome. Prophages also shape the phage susceptibility profiles of their hosts via encoded defense systems that protect against superinfection (19). Of additional interest is the plethora of plasmids identified in this study that do not have immediately apparent biological functions. These mobile elements are quite common across clinical isolates and may represent important contributors to Mab biology.

Bacteriophage-mediated transfer of genes between hosts and genomic rearrangements can result in mutual benefit for both bacterium and phage, but fundamentally, phages are pathogens. As a result, bacteria experience selective pressure to evade phage infection (20–22). This arms race between phage and host represents a second, more indirect method by which phages shape bacterial genomes. The selective pressures imposed on the bacterium can result in rapid evolution of a variety of components of the bacterial cell, especially surface-exposed components that serve as the entry point for phages (20). Preventing phage adsorption through modification of the cell envelope is the first line of defense and may be the most prevalent phage resistance mechanism (23). Adsorption requires conformational changes between multiprotein tail complexes and receptor proteins and sometimes relies on enzymatic processing (24). This multistep, dynamic process is tunable through mutation. Other bacteria exhibit mutations in phage receptor proteins, genes responsible for synthesizing those proteins, and pathways that secrete extracellular matrix; some bacteria even secrete competitive factors to obscure receptors (23). Surprisingly, mutants that clearly disrupt adsorption have yet to be isolated in mycobacteria. Moreover, the cell envelope components that function as receptors for mycobacteriophages are almost entirely unknown (25).

Dedrick et al. have revealed one clue that may help unravel the mystery of what dictates phage susceptibility in Mab. They find that solid-medium colony morphology (smooth versus rough) can predict susceptibility to phage and, perhaps, treatment outcomes. Smooth clinical isolates, which represent nearly 40% of the strains examined in this study, have limited susceptibility to phage, while rough strains are more vulnerable. The underlying genetics and lipid chemistry that separate the mucoid, smooth-edged morphotype from the dry, rough-edged morphotype are well documented (26). Smooth strains have more surface glycopeptidolipids (GPLs) that are produced by synthesis and transport genes including *mps1*, *mps2*, and *mmpL4*. A smooth-to-rough transition can be attained by mutating these genes, but it is not fully understood why cells convert *in vivo* and to what degree morphotype predicts pathogenicity. In general, the morphotypes seem to be equally represented in Mab patient populations. Rough mycobacterial variants have been described as more virulent and persistent (27, 28), although this is an area of ongoing study.

The dramatic difference in susceptibility between smooth and rough morphotypes warrants further examination of the mechanisms and determinants of phage adsorption. The loss of GPLs drives large-scale changes in the extracellular matrix that likely alter the accessibility of phage receptor epitopes, but these changes could also alter other processes such as phage dispersal. Mechanistic studies are needed to determine why the smooth morphotype is protected and whether this is consistent across mycobacterial pathogens. By examining strains that spontaneously developed phage resistance, Dedrick et al. identify a number of mutated genes in Mab that merit further study. Some of the mutations, such as those in *mps1* and a type I polyketide synthase (MAB_0939), likely generate resistance by altering surface composition and interfering with phage adsorption. Other mutations, such as the putative helicase, UvrD2, and the RNA Polymerase Omega subunit, rpoZ, require further mechanistic study but may impact other aspects of the phage life cycle. Viable mutations that generate resistance are relatively rare, occurring in only ∼40% of susceptible strains challenged with a single phage. This bodes well for phage therapies against Mab, as the acquisition of resistance by other species of bacteria against single phages is more common in clinical studies (29). Given that mycobacteria have a distinct cell envelope architecture and chemical signature (30), it is possible that mutational pathways that disrupt adsorption often undermine the integrity of the cell wall and are less permissive. If this is true, one might expect phage therapies to be complementary to antibiotics that target the mycobacterial cell wall.

These observations suggest novel combinatorial therapeutic approaches using drugs that influence cell wall composition—even if they are unable to kill by themselves—and phages, which target specific morphologic phenotypes. By inhibiting enzymes that produce key cell wall molecules or the transporters that direct molecules to the exterior of the cell, we may be able to both sensitize previously resistant bacteria and prevent the development of resistance to mycobacteriophage-mediated killing. The highly conserved MmpL transporter family, which has been studied extensively as it harbors promising antitubercular drug targets (31–33), may be a low-hanging example. Chemical inhibition of MmpL4 in Mab is likely to both inhibit bacterial growth and drive the transition from smooth to rough to synergize with phage therapies.

Identification of host factors that are mutated *in vitro* to generate resistance may provide a blueprint for how bacteria can evade administered phage therapies. But, it is important to recognize that mutants that thrive in the absence of immunity and other host pressures may not coincide with those that emerge in patients. A better understanding of resistance mechanisms seen in the growing number of case studies and clinical trials is necessary. This information may allow for the development of “off the shelf” antibiotic-phage combinations that can be administered at the onset of diagnosis. So, perhaps the answer to the difficult problem of Mab infections isn’t a choice between drugs and phages; it’s a combination of both.
